# Literature review: spectral imaging applied to poultry products

**DOI:** 10.1016/j.psj.2020.04.013

**Published:** 2020-04-26

**Authors:** Anastasia Falkovskaya, Aoife Gowen

**Affiliations:** UCD School of Biosystems and Food Engineering, University College Dublin, Belfield, Dublin 4, Ireland

**Keywords:** hyperspectral imaging (HSI), poultry, product quality, food safety, literature review

## Abstract

Consumption of poultry products is increasing worldwide, leading to an increased demand for safe, fresh, high-quality products. To ensure consumer safety and meet quality standards, poultry products must be routinely checked for fecal matter, food fraud, microbiological contamination, physical defects, and product quality. However, traditional screening methods are insufficient in providing real-time, nondestructive, chemical and spatial information about poultry products. Novel techniques, such as hyperspectral imaging (**HSI**), are being developed to acquire real-time chemical and spatial information about products without destruction of samples to ensure safety of products and prevent economic losses. This literature review provides a comprehensive overview of HSI applications to poultry products. The studies used for this review were found using the Google Scholar database by searching the following terms and their synonyms: “poultry” and “hyperspectral imaging”. A total of 67 studies were found to meet the criteria. After all relevant literature was compiled, studies were grouped into categories based on the specific material or characteristic of interest to be detected, identified, predicted, or quantified by HSI. Studies were found for each of the following categories: food fraud, fecal matter detection, microbiological contamination, physical defects, and product quality. Key findings and technological advancements were briefly summarized and presented for each category. Since the first application to poultry products 20 yr ago, HSI has been shown to be a successful alternative to traditional screening methods.

## Introduction

Many people rely on poultry products as a source of proteins, essential amino acids, mineral salts, and vitamins (Rouger et al., 2017). As demand for poultry products increases worldwide, producers need to ensure consumer safety and meet quality standards. Poultry products must be routinely checked for food fraud, fecal matter, microbiological contamination, physical defects, and product quality. Traditional screening methods (e.g., high-performance liquid chromatography, mass spectroscopy) are time-consuming and expensive and require sample destruction, and currently, most nondestructive spectroscopic methods are not totally representative of samples and unable to show spatial information (Gowen et al., 2007). Furthermore, conventional imaging techniques are not capable of providing information about the chemical composition of products. Novel imaging techniques, such as hyperspectral imaging (**HSI**), are being developed and used to acquire real-time information about products without destruction of sample to ensure safety of products and prevent economic losses.

### What is HSI?

**Figure 1:**
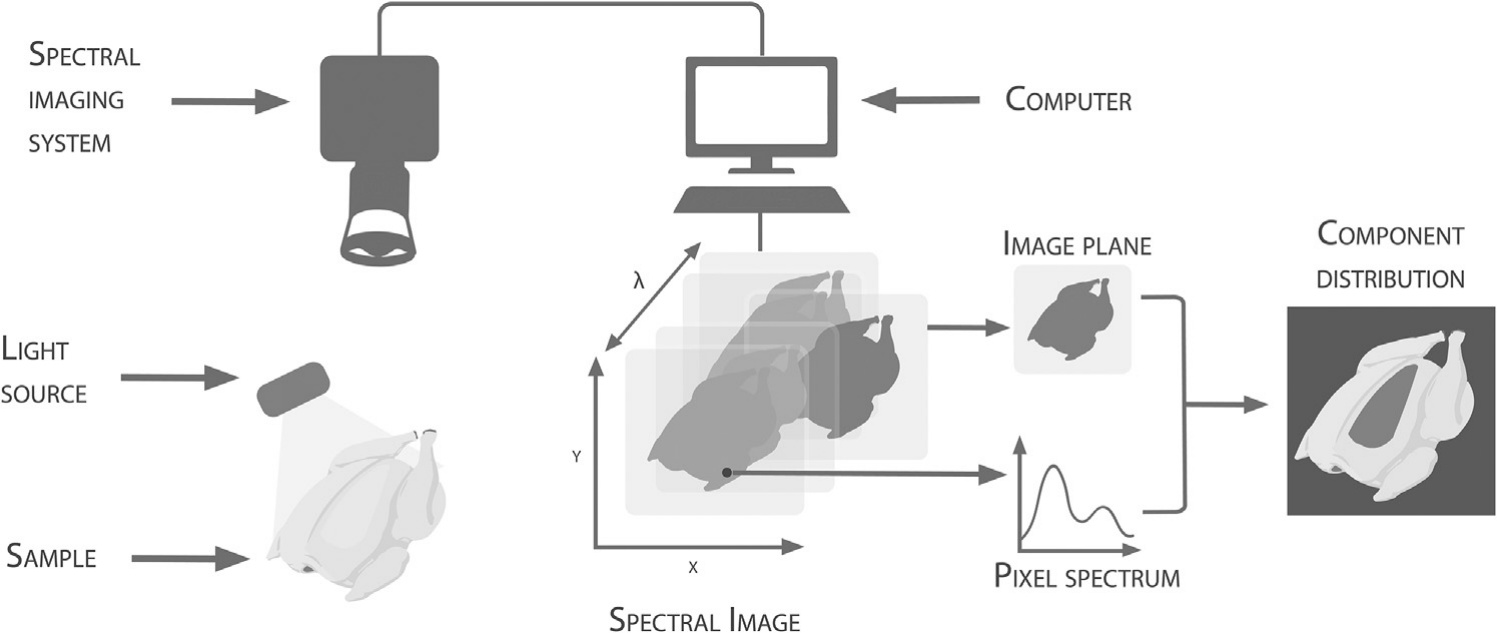
Schematic of typical HSI system hardware and workflow adapted from Boziaris (2014).

HSI is a nondestructive imaging technique that combines conventional imaging and spectroscopy to obtain both spatial and spectral information about an object (Gowen et al., 2007). Conventional imaging provides spatial information about an object but cannot provide any information about components that absorb or scatter light in wavebands other than RGB. On the other hand, spectroscopy can provide information about the chemical composition of an object at chosen wavebands but cannot provide spatial information about the object. By combining the 2 techniques, it is possible to give spectral information a spatial dimension. Assigning spatial dimensions to spectral data makes it possible to map chemical components of a sample, which is useful for nonhomogenous samples. This is done by creating a 3D block of data (i.e., a spectral image cube), with each slice representing the image at a certain waveband (Figure 1).

Resulting images are very data rich, with each pixel represented by a spectral image containing spectral information for the chosen range of wavelengths. The goal of HSI data processing is to reduce the dimension of data and retain enough to classify and quantify important regions of a sample (Gowen et al., 2007). Commonly used methods for classification of pixels can be grouped into either unsupervised or supervised. In both unsupervised and supervised methods, acquired images are divided into a training set and test set of images. The success of the method is determined by the accuracy with which the model created from the training set can predict characteristics in the independent test set. Unsupervised methods (i.e., principal component analysis [**PCA**]) do not rely on prior knowledge of pixel classes. Instead, unsupervised methods rely on software to classify pixels based on similarities in spectral characteristics determined by algorithms. In contrast, supervised methods (i.e., partial least squares discriminant analysis) do require intervention to select pixels representative of each class by hand from a training set.

The basic hardware required for HSI acquisition consists of a light source, lens, camera, spectrograph, and computer (Figure 1). Systems collect spectral information within defined wavelength ranges in the electromagnetic spectrum. Commonly used ranges include ultraviolet 100–400 nm, visible (**Vis**) 400–700 nm, near-infrared (**NIR**) 700–1,400, and short-wave infrared 1,400–3,000 nm. Spectral resolution of the system is the ability of the sensor to define between intervals in the chosen wavelength range. Spatial resolution is the physical area a pixel represents. A method for significantly increasing spatial resolution is by using hyperspectral microscope imaging (**HMI**). This is done by combining HSI with a microscope to increase resolution (Park et al., 2017). As a result, HSI has the potential for application at both the macroscopic and microscopic levels.

## Methods

The studies used for this review were found using the Google Scholar database by searching the following terms in combination: “poultry” and “hyperspectral imaging”. Commonly used abbreviations can be found in Table 1.

**Table 1:** Common abbreviations of data analysis methods terms used in this literature review.

Abbreviation	Full term
ABB	Adaptive branch and bound algorithm
ACO	Ant colony optimization
ANN	Artificial neural network
ANOVA	Analysis of variance
BPANN	Back propagation artificial neural network
CARS	Competitive adaptive reweighed sampling
FD	First derivative
FLDA	Fisher linear discriminant analysis
GLCM	Gray level co-occurrence matrix
GLM	General Linear Model
kNN	k-nearest neighbor classification
LDA	Linear discriminant analysis
MC	Mean centring
MLF	Multiple level data fusion
MSC	Multiplicative scatter correction
PCA	Principal component analysis
PLS-DA	Partial least square discriminant analysis
PLSR	Partial least squares regression
QDA	Quadratic discriminant analysis
RBF-SVM	Radial basis function - support vector machine
RC-PLSR	Regression coefficients - partial least squares regression
RMSE	Root mean squared errors
RMSEP	Root mean squared errors by prediction
SAM	Spectral angle mapper
SD	Second derivative
SIMCA	Soft independent modelling of class analogy
SNV	Standard normal variate
SNVD	Standard normal variate and detrending
SPA	Successive projections algorithm
STLR	Single-term linear regression
SVM	Support vector machine

After all relevant literature was compiled, studies were categorized into the following groupings based on their subject matter: fecal matter, food fraud, physical defects, microbiological contamination, and product quality.

## Results and discussion

Over the past 20 yr, a total of 67 studies have been published on applications of HSI to poultry products. Overall, the highest number of studies has been published in application to fecal matter (n = 20) and microbiological contamination (n = 20), followed by product quality (n = 13), physical defects (n = 10), and food fraud (n = 4) (Table 2). A summary of methods used by each study can be found in Table 2.

**Table 2:** Overall summary of all compiled studies by category.

Group	Citations	Number of studies	Acquisition mode (# of studies)	Spectral range (# of studies)	Classification method (# of studies)
Food fraud	(Garrido-Novell et al., 2018^1^; Kamruzzaman et al., 2014^2^; Oh et al., 2017^3^; Xiong, Sun, Pu, Zhu and Luo, 2015^4^)	4	Reflectance (4)^1,2,3,4^	NIR (1)^3^ Vis-NIR (2)^2,4^ SWIR (1)^1^	Decision tree (1)^1^ PLS (1)^3^ PLS-DA (2)^1,4^ PLSR (1)^2^
Faecal matter	(Cho et al., 2007^1^; Cho et al., 2005^2^; Heitschmidt et al., 1998^3^; Heitschmidt et al., 2007^4^; Lawrence et al., 2007^5^; Lawrence et al., 2003^6^; Lawrence et al., 2004^7^; Lawrence et al., 2006^8^; Liu et al., 2003^9^; Park et al., 2005, Park et al., 2006^10,11^; Park et al., 2010^12^; Park et al., 2002^13^; Park, Windham, Lawrence and Smith, 2007^14^; Park, Yoon, Lawrence and Windham, 2007^15^; Qin et al., 2011^16^; Windham, Heitschmidt, Smith and Berrang, 2005^17^; Windham, Smith, Berrang, Lawrence and Feldner, 2005^18^; Windham et al., 2003^19^; Yoon et al., 2011^20^)	20	Fluorescence (2)^2,16^ Reflectance (19)^1,2,3,4,5,6,7,8,9,10,11,12,13,14,^ ^15,17,18,19,20^	Vis (1)^16^ Vis-NIR (19)^1,2,3,4,5,6,7,8,9,10,11,12,13,14,15,17,18,19,20^	Band ratio (16)^2,3,5,7,8,9,10,11,12,13,14,17,18,19,20^ Decision tree (1)^4^ One-way ANOVA (1)^1^ PCA (7)^3,5,6,9,10,16,17^ PLSR (2)^6,17^ SAM (1)^15^ STLR (1)^4,17^
Microbiological contamination	(Chao et al., 2007^1^; Chao et al., 2008^2^; Y. Z. Feng et al., 2012^3^; Feng and Sun, 2013, Feng and Sun, 2012^4,5^; Jun et al., 2010^6^; Jun et al., 2009^7^; Jun et al., 2008^8^; Lu and Chen, 1999^9^; Park et al., 2011^10^; Xiong, Sun, Pu, Xie, et al., 2015^11^; C. C. Yang, Chao and Kim, 2009^12^; Ye et al., 2016^13^; Yoon et al., 2010^14^, 2009^15^, 2012^16^, Eady et al., 2019^17^, Eady et al., 2015^18^, Park et al. 2015^19^, Park et al., 2017^20^)	23	Fluorescence (3)^6,7,8^ Reflectance (12)^1,2,3,4,5,9,11,12,13,14,15,16^ Transmittance (1)^10,17,18,19,20^	Vis (3)^6,7,8^ Vis-NIR (11)^1,2,9,10,11,12,13,14,15,16,17,18,19,20^ SWIR (3)^3,4,5^	ANOVA (2)^8,18^ Band ratio (3)^6,11,12,14,16^ Fuzzy logic (2)^1,2^ kNN (2)^20^ LDA (2)^19,20^ Mahalanobis distance (2)^19,20^ PCA (9)^6,7,8,13,15,16,18,20^ PLS-DA (1)^20^ PLSR (5)^3,4,5,18^ QDA (3)^17,19,20^ Qualitative analysis (2)^9,10^ SIMCA (1)^18^ SVM (2)^17,19,20^
Physical defects	(Chao et al., 2002^1^; Du et al., 2007^2^; Fletcher and Kong, 2003^3^; I. Kim, Kim et al., 2004^4^; T. Kim et al., 2010b^5^; Kong, 2003^6^; Kong et al., 2004^7^; Nakariyakul and Casasent, 2004, Nakariyakul and Casasent, 2009^8,9^; Yoon et al., 2008^10^)	10	Fluorescence (6)^2,3,45,6,7^ Reflectance (4)^1,8,9,10^ Transmittance (1)^10^	Vis (7)^2,3,4,5,6,7,8^ Vis-NIR (3)^1,9,10^	ABB algorithm (1)^9^ Fuzzy logic (4)^1,4,6,7^ LDA (1)^5^ PCA (5)^1,3,7,8,10^ SVM (1)^2,3^
Product quality	(Elmasry et al., 2010^1^; Iqbal et al., 2013^2^; Jia et al., 2017^3^; Jiang, Yoon, Zhuang, Wang, Lawrence, et al., 2018^4^; Jiang, Yoon, Zhuang, Wang, Li, et al., 2018^5^; Kandpal et al., 2013^6^; Khulal et al., 2016^7^; Khulal et al., 2017^8^; Xiong et al., 2014^9^; Xiong, Sun, Xie, Han and Wang, 2015^10^; Y. Yang, Wang et al., 2018^11^; Y. Yang, Wang et al., 2018^12^; Yoon et al., 2016^13^)	13	Reflectance (13)^1,2,3,4,5,6,7,8,9,10,11,12,13^	NIR (2)^1,2^ Vis-NIR (10)^3,4,5,6,7,8,9,10,12,13^ SWIR (1)^11^	ACO (2)^7,8^ ANN (1)^9^ LDA (1)^13^ PCA (7)^1,2,3,4,5,6,13^ PLS-DA (1)^4^ PLSR (7)^3,4,5,9,10,11,12^
Total		67	Fluorescence (11) Reflectance (53) Transmittance (5)	NIR (3) Vis (11) Vis-NIR (48) SWIR (5)	ABB algorithm (1) ACO (2) ANN (1) ANOVA (2) Band ratio (19) Decision tree (2) Fuzzy logic (6) kNN (2) LDA (4) Mahalanobis distance (2) One-way ANOVA (1) PCA (28) PLS (1) PLS-DA (4) PLSR (8) QDA (3) Qualitative (2) SIMCA (1) STLR (1) SVM (2)

Superscripts show how particular citations are linked to entries in the the other columns of the table (i.e., acquisition mode, spectral range, and classification method).

### Fecal Matter

Minimizing fecal contamination of poultry products minimizes the spread of bacterial pathogens. As a result, it is vital for processors to detect and remove any fecal contamination on poultry products to comply with regulations and prevent the spread of bacteria such as *Salmonella*, *Campylobacter*, and *Escherichia coli*. In addition, any poultry-processing surfaces must be kept contaminant free to prevent spread of bacterial pathogens. Traditional detection methods rely on the use of human inspectors to detect visible fecal contamination in-line, which is tedious and prone to human error (Park et al., 2002). Because of the high speed of modern production lines, human inspectors are unable to check each bird. HSI has been shown to be an efficient alternative for in-line inspection of poultry products, capable of high-throughput detection at high speeds.

A total of 20 studies have been published on applying HSI to detection of fecal matter. This is the first application of HSI to poultry products. All 20 studies used line-scanning acquisition mode. A common challenge for applying HSI to detection of fecal matter is that fecal material can vary in color, consistency, and composition based on source (e.g., duodenum, ceca, colon) and feed type. In addition, fecal matter may be obscured by shadows of the carcass.

The first study to apply HSI to fecal matter detection was carried out by Heitschmidt et al. (1998). The study confirmed a visual difference between the spectra of meat and feces on whole chicken carcasses and considered several data analysis protocols for future quantitative detection (e.g., PCA, Minimum Distance, Mahalanobis Distance, and Spectral angle mapper [**SAM**]). Of the proposed data analysis protocols, PCA was cited as potentially the most powerful method; however, it was limited by processing time required to work with spectral images. Less than a decade later, PCA was successfully applied to compare Vis-NIR spectroscopy and HSI for detection of fecal contamination (Lawrence et al., 2004). For spectroscopic analysis, samples were prepared by placing either pieces of chicken breast skin or feces into sample cells with a quartz optical surface. For HSI, whole broiler carcasses were imaged either uncontaminated or with feces applied. This study showed that HSI has the capacity to create models using less intensive sample-preparation protocols and higher accuracy than traditional spectroscopic methods.

Many of the following applications were done by groups from the USDA. Predominant groups include Environmental Microbial and Food Safety Laboratory (USDA-ARS), Instrumentation and Sensing Laboratory (USDA-ARS), and the Richard B. Russell Research Centre (USDA-ARS). One of the most important studies to apply HSI to fecal matter detection identified optimal wavelengths corresponding to fecal contamination that could be used in a band ratio to classify pixels (Park et al., 2002). The significance of this particular study is that the developed band ratio was later used, validated, and built on by 13 further studies applying HSI to fecal detection (Lawrence et al., 2003, Windham et al., 2005b, Windham et al., 2005a, Lawrence et al., 2006, Lawrence et al., 2007, Windham et al., 2003, Park et al., 2005, Park et al., 2006, Park et al., 2007b, Park et al., 2007a, Park et al., 2010, Heitschmidt et al., 2007, Yoon et al., 2011). Images were obtained of pure fecal material from broilers fed one type of feed (i.e., corn or soybean meal), uncontaminated broiler carcasses, and fecal material on broiler carcasses. Using different combinations of the optimal wavelengths determined from PCA, the band ratio of 565/517 nm was developed. Lawrence et al. (2003) provided further justification for the band ratio by relating the wavelength at 565 nm to myoglobin and hemoglobin of the breast skin and the wavelength at 517 nm to color differences between feces or ingesta and breast skin. When tested on fecal material from broilers fed different diets (corn and soybean, milo and soybean, wheat and soybean), the band ratio was slightly altered from 565/517 nm to 574/588 nm (Windham et al., 2003). The wavelength of 588 nm is better suited to detection as it represents a color that is common between fecal material from broilers fed different diets. Further refinement of the band-ratio technique included addition of a third wavelength (802 nm) to help classify problematic pixels in images (Windham et al., 2005a), preprocessing methods such as calibration and smoothing (Park et al., 2006), determining ideal thresholds for image processing (Windham et al., 2005b), dynamic thresholding techniques (Park et al., 2007b), hardware updates (e.g., new camera, new spectrograph, improved lighting) (Heitschmidt et al., 2007), and testing compatibility with LED lighting (Lawrence et al., 2007). In addition, the band ratio method was shown to be successful in real-time detection of fecal contaminants on whole chicken carcasses (Park et al., 2010) and even at commercial production line speeds of 140 and 180 birds per minute (Yoon et al., 2011). In only 20 yr, HSI has gone from visually looking at differences between spectra to demonstrating the feasibility of using HSI systems for high-speed in-line detection of fecal contamination on poultry products while maintaining high detection accuracy.

A potential problem for the band ratio technique is that postwash stains are classified as contamination by the algorithms. However, stains are not the same as actual fecal contamination and are not usually considered to be contaminants (United States Department of Agriculture, 1998). The band ratio was used to assess fecal contamination on birds before and after washing in a production line (Lawrence et al., 2006). A pilot scale bird washer was created for the purpose of this experiment. Fecal material was applied on to clean whole bird carcasses, after which whole birds were imaged, washed, and imaged again. The imaging system identified 98% of contamination before washing. After washing, the system detected 45% of cecal and 35% of duodenum stains. Future systems need to account for this fact and ensure that HSI systems are detecting true contamination and not stains.

Several alternative methods to the band ratio have been proposed for detecting fecal contamination on poultry products. The goal of searching for an alternative ratio was to minimize false-positive errors and misclassification errors associated with the band ratio. Cho et al. (2005) used different band ratios to detect diluted fecal residue on poultry-processing equipment using both reflectance and fluorescence modes of HSI. Samples were obtained by diluting poultry fecal and ingesta residue to 1:5, 1:10, 1:15, 1:50, and 1:100 by weight and imaged on stainless steel. Fecal residue could be best detected with 97.2% accuracy in the most diluted samples (1:100) using fluorescence mode, using a ratio of 482/553 nm. The high classification rate for diluted samples shows potential for accurate detection at very low concentrations of contaminants. However, future work is needed to determine the actual limit of detection of fecal contaminants by HSI.

Another alternative to the band ratio was using SAM algorithm to classify type and source of fecal contaminants (Park et al., 2007a). Whole broilers were imaged before and after ingesta and fecal contaminants were applied to the carcasses. Although using the SAM algorithm resulted in high accuracy, it was not as good as the band ratio. The most recent study to apply HSI to fecal detection aimed to detect and classify any organic residues (e.g., fat, blood, feces, and ingesta) on poultry-processing equipment (e.g., stainless steel) using fluorescence HSI (Qin et al., 2011). A 4-class (“stainless steel”, “fat”, “blood”, and “feces”) SIMCA model to distinguish between different types of organic matter could do so with 97.5% accuracy. The high accuracy of classification is promising for future application of HSI to detecting and discriminating between different classes of contaminants on poultry-processing equipment.

### Food Fraud

Food fraud is the intentional deception of consumers through adulteration of products in pursuit of financial gain (EU Regulation No. 1169/2011 of 25 October 2011 on the provision of food information to consumers). In the case of meat products, food fraud can occur by either mislabelling what animal the meat came from, addition of various “filler” substances, mislabelling of any process the meat underwent, or mislabelling the quality of the animal's welfare. Producers may be motivated by financial gain to label cheaper alternatives as high-quality products, violating the right of consumers to information about their food. A variety of traditional methods have been developed to detect food fraud: molecular techniques, chromatography, isotopic techniques, spectroscopy, sensory analysis, and immunological assays (Danezis et al., 2016). Traditional methods used in food fraud detection are time-consuming, labor-intensive, tedious, and require sample destruction (Kamruzzaman et al., 2014). Because the definition of food fraud is so wide-ranging, it is difficult to develop a single technique robust enough to detect all types of food fraud. As a result, no standardized method exists to ensure the EU Regulation is being met. HSI could be used as an alternative to traditional methods for standardized mass scale detection of food fraud.

To date, only 4 studies have focused on detecting food fraud in poultry products using HSI. All studies have used line-scanning acquisition with reflectance mode. Types of food fraud researched include chicken food fraud in minced beef (Kamruzzaman et al., 2014), mislabelling of broilers as free-range chickens (Xiong et al., 2015b), differentiating between pork and poultry meat and bone meal (MBM) (Oh et al., 2017), and discriminating between processed pork, poultry, and fish proteins (Garrido-Novell et al., 2018).

The first study to apply HSI to detecting food fraud related to poultry products intended to detect chicken food fraud in minced beef (Kamruzzaman et al., 2014). Detecting food fraud in minced meat is particularly difficult, as mincing destroys any distinguishable morphological characteristics. As a result, it is relatively easy for producers to use less expensive meats as a filler in their product. Samples were created by mixing minced beef samples with 0–50% minced chicken at 2% intervals. The Partial least squares regression (PLSR) model built on the absorbance spectral profile was found to be the best model for detecting food fraud in minced beef. The PLSR model was repeated using only 5 optimal wavelengths, and prediction maps were created based on the identity of each pixel. The methods presented by this study were successful in detecting and visualizing chicken food fraud in minced beef, showing potential for widespread implementation.

One year later, HSI was applied to differentiate between free-range and broiler chicken meats (Xiong et al., 2015b). Free-range chickens are defined as slow-growing and raised with access to outdoor areas. In blind taste tests, consumers cannot easily distinguish the difference between free-range and broiler chickens based on sensory characteristics (Lawlor et al., 2003, Castellini et al., 2008). This means consumers are not choosing to buy free-range chickens solely based on taste but also because of the welfare conditions chickens are raised in. Because consumers have a difficult time distinguishing chickens based on sensory characteristics, producers may be motivated to mislabel broiler chickens as free-range chickens for financial gain. However, the 2 types of chickens differ in feed type and level beause of their difference in access to the outdoors. This fact made it possible to detect differences between free-range and broiler chickens (Xiong et al., 2015b). Samples were obtained by cutting slices of breast meat from free-range and broiler chicken carcasses and imaged in the Vis-NIR. After fusing spectral data selected by successive projections algorithm (SPA) and textural data selected by PCA followed by gray level co-occurrence matrix, an radial basis function–support vector machine model was found to be the best model for classification. The model was able to differentiate between free-range and broiler chicken meats with high accuracy.

Verifying what animal a meat product has come from is also important to prevent intraspecies recycling. EU Regulation (EC) No. 1774/2002 of 12 May 2003 currently prohibits intraspecies recycling in pigs (i.e., pigs cannot be fed pig by-products). Instead, poultry by-products can be used as feed to meet the demands of the regulation. Ensuring that this regulation has been met can be difficult, particularly because animal by-products used for feedstuffs rarely resemble any recognizable part of the animal it originally came from. Using MBM prevents food waste and is an economical option for feed; however, the animal it came from must be clearly identified to prevent intraspecies recycling. In a study by Oh et al. (2017), HSI was used to differentiate between poultry and pork MBM. Mixed samples of pork and poultry MBM were imaged and modeled using PLS, successfully showing that HSI is capable of identifying highly rendered meat origin with high accuracy.

Rendered meat by-products are not only for animal consumption. Processed animal protein is a category of rendered meat by-products that are considered fit for human consumption by EU Regulation (EC) No. 1069/2009 of 21 October 2009. The most recent study on determining what animal a meat product has come from uses processed animal protein to discriminate between pork, poultry, and fish proteins (Garrido-Novell et al., 2018). Individual samples in this study were not mixed before imaging (i.e., a poultry sample contained only poultry and poultry by-products and likewise for poultry and fish). After imaging, spectral and textural data were modeled using partial least square discriminant analysis (PLS-DA) and fused for species classification by classification trees. Compared with spectral PLS-DA models alone, fusion of data allowed for discrimination between processed pork, poultry, and fish proteins.

Most existing studies have focused on determining what animal the meat has come from, with one exception of detecting mislabelling of animal welfare. No work has been published on detecting filler substances or any processing the poultry products may have undergone (e.g., detecting previously frozen poultry products mislabelled as fresh). Future research and implementation of HSI in commercial testing could provide a standardized way to detect many different types of food fraud.

### Microbiological Contamination

Poultry products contaminated by the pathogens *Salmonella* and *Campylobacter* were found to be the most common causes of food-borne illness in people (EFSA and ECDC, 2016). If contaminated poultry products are not identified and removed from market, bacteria on food surfaces have the potential to spoil food and cause serious harm to consumers. A recent study found an overall 11.5% prevalence rate of contamination by *Staphylococcus aureus* in ready-to-eat and ready-to-cook poultry across various retail sources (Wang et al., 2018). The presence of contaminants at the retail level and persistence of food-borne illness outbreaks indicate that current screening methods are not adequate at completely preventing contaminated and poor-quality poultry products from entering the market. In addition, spoilage of poultry products due to bacteria contamination results in economic losses for producers and retailers (Rouger et al., 2017). HSI has been applied to detecting microbiological contamination in poultry products in a total of 20 studies, using line-scanning with fluorescence, reflectance, and transmittance modes. Detection of microbiological contaminants can be further subdivided into the following categories: symptoms of bacterial infection (septicaemia and toxaemia), bacteria on agar, bacteria on processing surfaces, bacteria on poultry products, and bacteria detection using HMI.

#### Symptoms of Bacterial Infection

Poultry carcasses showing symptoms of septicaemia and toxaemia must be detected and removed from the production line to prevent any food-borne illness in consumers (Fisher et al., 1998). Septicaemia is a systemic infection caused by the presence of pathogenic microbiological contamination in the bloodstream (Chao et al., 2007). Toxaemia is a localized infection caused by toxins or pathogenic microbiological contamination (Chao et al., 2007). Traditional detection methods rely on inspectors to visually check for infection symptoms on poultry carcasses (e.g., lesions, degeneration of skeletal muscles), as indicators of septicaemia or toxaemia (Fisher et al., 1998). Inspectors typically work at a rate of 35 birds per minute on processing lines (Yang et al., 2009). Because traditional methods rely on inspectors, the process is slow, tedious, and prone to human error.

The first study to attempt detection of septicaemia in poultry was carried out by Lu and Chen (1999), concluding that birds affected by septicaemia had visually distinct spectra from healthy birds. The next advancement was made by Chao et al. (2007), using fuzzy logic on selected wavelengths to classify if birds were affected by septicaemia and toxaemia. In the following year, the same group successfully applied the automation methods to freshly slaughtered whole chicken carcasses on a high-speed production line for high-throughput detection with 96% accuracy at a speed of 140 birds per minute (Chao et al., 2008). The most recent study on septicaemia and toxaemia detection combined HSI with multispectral imaging (Yang et al., 2009). A band ratio capable of septicaemia and toxaemia detection was determined using HSI, after which multispectral imaging was used to obtain images containing spectral information for only the key wavelengths. The system could easily switch between hyper and multispectral modes without the need for recalibration. When applied to a high-speed production line at a speed of 140 birds per minute, this method identified infected birds with the same accuracy as human inspectors. The combined results of these 4 studies indicate that HSI can be used to successfully detect septicaemia and toxaemia. Furthermore, HSI can be successfully used in high-speed production lines for automated detection of infection and reduction of human error.

#### Bacteria on Poultry Products

Traditional methods of measuring bacterial contamination on poultry products rely on collecting bacteria from a point sample of product. This is problematic, as it does not give an accurate representation of what is happening on the whole product. To date, 4 studies have attempted to use HSI to detect bacteria directly on poultry products. All studies have been laboratory based, with no attempts at high-throughput processing line application.

The first study to apply HSI to detect bacteria on poultry products aimed to predict natural *Enterobacteriaceae* loads on chicken fillets (Feng et al., 2012). Chicken fillet samples were acquired at a grocery store and refrigerated at 4°C. For a total of 9 D, one package was taken to be imaged each day. Samples slices were flattened in a petri dish and imaged in the NIR spectral range. After imaging, reference *Enterobacteriaceae* loads were determined by homogenizing and plating the samples on agar for colony counting after incubation. Using PLSR, the model was capable of successfully predicting natural *Enterobacteriaceae* loads on chicken fillets. The same group predicted natural *Pseudomonas* loads on chicken fillets (Feng and Sun, 2013). Similar experimental methods were used as for the previous study; however, samples were imaged every 12 h for the last 3 D of the 9-D experiment. A PLSR model created to predict *Pseudomonas* loads using 14 selected optimal wavelengths resulted in the best prediction model.

Another application of HSI is to predict total viable count (TVC) of bacteria naturally occurring on chicken breast fillets (Feng and Sun, 2012). TVC is a measure of the approximate bacteria concentration and can be used as an indicator of bacterial spoilage. Samples were acquired from a local store and refrigerated at 4°C until obvious signs of spoilage were observed (e.g., slime and smell). Samples were cut and imaged over 9 D using the procedure as in the study by Feng and Sun (2013). A PLSR model built on the absorbance spectral profile was found to be better at predicting TVC than models built using reflectance or Kubelka-Minck spectral profiles. A more recent study also used HSI to predict natural TVC counts on chicken breast fillets (Ye et al., 2016). Samples were acquired from one batch at local supermarket, stored at 4°C, and imaged every 24 h for 11 D. This study proposed a “two-band freshness index” to predict TVC loads using just 2 wavelengths, determined by correlation coefficients. Two-band freshness index is proposed as a more efficient method than PLSR models, as less spectral data are required to build the model. However, the R^2^ of 0.68 was much lower than that given by PLSR models developed by Feng and Sun (2012).

Most studies attempting to detect bacteria directly on poultry products have used chicken breast fillets as samples. However, it should be noted that bacteria are most commonly found on poultry skin rather than on meat (Rouger et al., 2017). Future work is needed to successfully detect bacteria on poultry skin. This could be challenging, as bacteria can be harbored by feather follicles and pass undetected.

#### Hyperspectral Microscope Imaging

The following 4 studies used HMI to image samples with staring-face mode and transmittance illumination in the Vis-NIR range. All 4 studies were carried out by the same group at the USDA Richard B. Russell Research Centre using the same equipment. These studies are unique from previous applications because they include the use of a microscope to allow for bacterial cell-level imaging. In addition, the instrumentation includes acousto-optic tunable filters for staring-face imaging, which allows for image acquisition without movement of the sample or apparatus.

The first study to apply HMI to microbiological contamination in poultry was conducted by Park et al. (2011) to determine feasibility and optimal parameters (integration time and gain) for detection of Shiga Toxin–producing *E. coli* and *Staphylococcus enteritidis* biofilms. A Center for Disease Control biofilm reactor was inoculated with 10 mL of 10^8^ CFU/mL *S. enteritidis* at 36°C in a Trichoderma-selective agar medium for 24, 48, and 72 h. Stainless steel coupons were suspended in the reactor and taken out for imaging every 24 h. Initial concentration, incubation time, and inoculation procedures were not made explicit for *E. coli* biofilms. After imaging, spectra were examined visually for differences between the 2 types of bacteria. This preliminary study concluded that *E. coli* and *S. enteritidis* had distinct spectral profiles that could be used to create spectral fingerprints for rapid bacterial identification in the future. Spectral profiles from *E. coli* could be characterized by increased intensity at 546 nm. In addition, biofilms formed by *S. enteritidis* could be characterized by increased intensity at 498, 522, 550, and 594 nm. Park et al. (2011) note that future work needs to develop standard calibration protocols and could use multiple acquisitions to reduce noise. However, limitations do exist when applying HMI to imaging live cells. Because live cells are motile, blurring can occur in images because of cell movement. To apply HSI to detection of bacteria on a mass scale, future work needs to develop techniques of coping with cell movement in images.

The next study to apply HMI to microbiological contamination focused on discriminating between gram-positive (i.e., *Salmonella*) and gram-negative (i.e., *Staphylococcus*) bacteria on Brilliant Green Sulfa (BGS) agar plates (Park et al., 2015). Gram-negative bacteria structurally differ from gram-positive bacteria because they have an additional outer membrane. Five serotypes of *Salmonella* and 5 species of *Staphylococcus* were grown in Tryptic Soy Broth for 18–24 h, diluted in a serial dilution to 10^−6^, plated on BGS agar, and incubated for 24 h at 35°C. Then, 3 μL of bacterial solution was resuspended in deionized water and placed on a glass microscope slide. Before image acquisition, cells were immobilized using a modified drying method to prevent image blurring. *Staphylococcus* had higher variability in peak intensity than *Salmonella*, possibly because of the outer membrane present in gram-negative *Staphylococcus* scattering more light than the inner membrane. Five different classification methods (Mahalanobis distance, k-nearest neighbour classification, linear discriminant analysis [LDA], quadratic discriminant analysis [QDA], and SVM) were compared for their ability to discriminate between bacteria types, determining that SVM is the best method for discriminating between gram-positive and gram-negative bacteria because it has the highest accuracy rate of 99.99%. This study is an important milestone because it successfully determines the spectral profiles of bacteria based on spatially distinct components within one cell (inner and outer membrane).

The next study applied HMI to detection of *Salmonella* serotypes at different incubation times with HMI (Eady et al., 2015). Samples were prepared by obtaining 5 different *Salmonella* serotypes from chicken rinsates. The same methods were used for inoculation as in the study by Park et al. (2015), with altered incubation times of 8, 10, 12, or 24 h. Before imaging, bacteria was resuspended in sterile water, and 3 μL of this mixture was placed on a microscope slide, air-dried, and covered with a cover slip. By imaging bacteria at different incubation times, cells were captured at various life cycle stages, capturing variation in scattering patterns due to incubation time. Eady et al. (2015) were able to classify *Salmonella* serotypes using SIMCA with equal accuracy at 8 and 24 h, showing the potential of HMI for rapid and early detection of bacteria.

Subsequently, Park et al. (2017) carried out a study to determine which classification method (PLS-DA, k-nearest neighbor classification, LDA, QDA, and SVM) is most suitable for distinguishing between the *Salmonella* serotypes used by Eady et al. (2015). Samples were prepared using the same methods as in the study by Park et al. (2015). The study concluded that SVM is the best method for discriminating between gram-positive serotypes. Being able to accurately discriminate between bacterial serotypes is important for the food industry, as not all serotypes are harmful to human health. In addition, serotype identification is used to trace outbreaks of *Salmonella* to their source (CDC, 2015).

The most recent study compared *Salmonella* detection in chicken rinsate using HMI vs. real-time PCR (Eady et al., 2019). Samples were prepared from both pure stock cultures of *Salmonella typhimurium* and rinsates of prechilled chicken carcasses. Stock cultures were inoculated into Tryptic Soy Broth overnight at 35°C, centrifuged, resuspended as pellets, and added to water until a concentration of 10^9^ CFU/mL was reached. Rinsates from 2 chicken carcasses were additionally inoculated to a final concentration of 100 CFU and incubated overnight. One rinsate was preserved as natural fauna. BGS Agar plated with 10^−5^ dilutions was incubated overnight at 35°C resulting in 25–250 CFU/plate. A total of 70 *Salmonella* and non-*Salmonella* colonies were selected and imaged, by resuspending single colonies in sterile water, pipetting 3 μL of that on a microscope slide, air drying, and covering with a cover slip. Samples were then classified at the cell level using QDA and cross-validated. Classification results were then compared with reference results from real-time PCR, performed using same colonies. *Salmonella* could be detected with up to 98.5% accuracy. Classification using HMI was faster than traditional methods. It took 2 D to classify bacteria using real-time PCR and only 1 D using HMI. The significance of this study is that it directly compared HMI with the current standard of detection (PCR), showing rapid and accurate classification is possible using HMI. For this to become a reality, future work needs to be done to create an exhaustive collection of bacterial fingerprints to create a robust classification resource that accounts for more bacterial variation.

To date, all reported HMI attempts have dried samples on microscope slides before imaging, to immobilize bacteria. Although this technique is possible using slides, it would not be suitable for attempting to detect bacteria directly on poultry products in a nondestructive way or on processing equipment in a real processing setting.

### Physical Defects

For the purposes of this literature review, physical defects are defined as physical irregularities in poultry products. While the other categories discussed herein focus on chemical characteristics and contamination, this category is mainly concerned with the detection of tumors on poultry skin and bone fragments. In total, 10 studies have focused on detecting physical defects in poultry products.

#### Tumors

Tumors on chicken skin are visible as round lesions surrounded by thickened skin and dermis (Chao et al., 2002). Tumors can be hard to detect because they are more characterized by shape distortion than discoloration (Kim et al., 2010). Despite no scientific evidence linking human consumption of tumors on chicken skin with any ill effects, any carcasses with tumors on the skin are condemned. HSI could be introduced to detect tumors on poultry products as an efficient alternative to human inspection.

The first attempt to detect tumors on chicken skin was made by Chao et al. (2002). To obtain samples, whole chickens were assessed by a veterinarian, and any chickens with tumors were marked. Then, line-scanning reflectance mode HSI was used to image whole carcasses in the Vis-NIR range. Using a fuzzy logic model to predict if pixels were tumorous, this method was able to correctly detect 91% of normal chicken skin and 86% of tumorous skin. The next 4 studies on tumor detection used flouresence mode HSI to detect tumors on whole carcasses using different data analysis methods including SVM (Fletcher and Kong, 2003) and fuzzy logic (Kong, 2003, Kim et al., 2004, Kong et al., 2004). All three studies using fuzzy logic suffered from high false-positive rates and were unable to detect small tumors in the early stage of development. The false-positive rate was reduced by a feature selection algorithm developed by Nakariyakul and Casasent (2004), using reflectance mode HSI images of whole chicken carcasses. Tumors are made up of 2 different portions—lesion and thickened skin. The feature selection algorithm developed in this study treats the tumor portions separately (i.e., lesions and thickened skin) and then fuses them to detect tumors. This resulted in fewer false-positives in detection. Other proposed feature-selection algorithms include recursive divergence (Du et al., 2007) and adaptive branch and bound algorithm algorithm (Nakariyakul and Casasent, 2009). Using thickened skin features along with lesion features reduced the number of false positives. The most recent application of HSI to tumor detection on poultry skin aimed to develop an optimal emmision filter for detection (Kim et al., 2010). Flourescence images taken using an excitation wavelength of 365 nm in whole chicken carcasses by Du et al. (2007) were used to create the emmision filter. A band-pass filter of 425–475 nm was determined to be the most appropriate, as it resulted in the highest contrast images. The method can be used to select significant wavelengths and provides continuous priority for those bands. Over the past 20 yr, substantial progress has been made in applying HSI to tumor detection in poultry products.

#### Bone Fragments

Purchasing deboned poultry products saves time and energy required to manually debone poultry. However, during processing, bone fragments can potentially be embedded into products. Presence of bone fragments in poultry products can be hazardous to human health and costly to processors and can result in a loss of customers (Smith, 2001). To prevent health hazards and reduce economic losses, products with embedded bone fragments must be detected and removed. The most common traditional method of screening for embedded bone fragments in poultry products is X-ray screening (Yoon et al., 2008). However, X-ray screening is ionising and prone to high false-positive rates (Yoon et al., 2008). Although HSI is considered a surface imaging technique, information about a sample can be obtained up to several millimetres deep depending on how far light can penetrate into the sample (Wu and Sun, 2013). The light penetration depth of poultry products has not yet been determined. In an attempt to create a more efficient nonionising system to detect embedded bone fragments in chicken breast fillets, Yoon et al. (2008) used a combination of reflectance and transmittance modes of HSI. Samples were then all compressed to a thickness of 1 cm and imaged in transmittance and reflectance modes. The combination of reflectance and transmittance light sources in image acquisition allowed for the detection of embedded bone fragments in chicken breast meat when the bone was embedded close to the surface. Transmittance spectra were too similar for meat and bone to be useful on their own. Detection accuracy was 50% for portions of bones fully embedded inside the chicken sample and 85% for portions of bone close to the skin using a nearest neighbor classifier. Unfortunately, this technique is also prone to a high false-positive rate. Because HSI is a surface imaging technique, detection of embedded bones in poultry products may not be a potential application. In addition, the methods used by this study relied on compression of meat, making this a destructive application of HSI.

### Product Quality

Consumers expect quality when purchasing poultry products. Inspectors rely on pH meters, conventional imaging, and destructive laboratory techniques to test product quality. However, quality is difficult to quantify, as it is subject to the judgement of inspectors. HSI could potentially be used to quantify desirable attributes and classify the quality of poultry products in a standardized way.

In total, 13 studies have attempted to apply HSI to determining the quality of poultry products. These studies can be subcategorized based on what metric they use to define quality: water content, pH, and color; chemical measures (e.g., total volatile basic nitrogen [TVB-N], 2-thiobarbituric acid reactive substances [TBARS]); and other sensory attributes (e.g., springiness, tenderness).

#### Water Content, pH, and Color

The first attempts to apply HSI to assessing quality in poultry products aimed to classify the quality of cooked sliced turkey hams (Elmasry et al., 2010, Iqbal et al., 2013). High-quality ham is injected with the lowest proportion of brine, whereas low-quality ham is injected with a high proportion of brine. In both studies, samples were prepared by injecting 4 different levels of brine (10, 20, 30, 40%) into 4 ham blocks, creating 4 quality levels of ham (premium, medium-high, medium-low, and low). In the first attempt, LDA was successfully used to classify the samples with an 100% classification rate and create classification maps (Elmasry et al., 2010). The later study expanded on these results by successfully predicting moisture, color, and pH of the turkey ham slices using PLSR models (Iqbal et al., 2013). The classifications made by the models matched those determined by traditional techniques, showing the ability of HSI to distinguish moisture, color, and pH of poultry products.

To study what quality factors are affected by postmortem deboning time, the following study predicted pH and color of deboned chicken breast fillets using Vis-NIR HSI (Jiang et al., 2018b). Samples were prepared by deboning chicken breast fillets at 2, 4, and 24 h postmortem. For comparison with traditional methods, color of the chicken breasts was measured using a spectrophotometer and pH using a pH probe. All reference and HSI measurements were acquired 24 h postmortem. Using an LDA model, differences between deboning times were only found for color and not pH. PLSR models were successful in accurately predicting both pH and color values. Further advancements were made by Yang et al. (2018a) by comparing Vis-NIR and NIR HSI for determining chicken breast fillet color, pH, moisture, drip loss, and salt-induced water gain. Whole chicken breast fillets were obtained directly after chilling and transported to the laboratory within 15 min. The exact time of imaging postmortem was not made explicit. By comparing PLSR models, Vis-NIR performed better for color and pH determination, whereas NIR performed better in drip loss, expressible fluid, and salt-induced water gain determination. Moisture determination was not successful by either system. Overall, these studies showed that HSI is capable of predicting pH and color of poultry products with reasonable accuracy. Although color can be predicted using less sophisticated methods, HSI allows for the simultaneous prediction of several attributes using one instrument.

#### Chemical Measures

Certain chemical measures can be used as predictors of quality and markers of spoilage. Chemical measures of importance in poultry product quality assessment include TBARS, hydroxyproline, and TVB-N.

The first study to attempt this predicted TBARS content in chicken meat, as an indicator of freshness (Xiong et al., 2015a). TBARS is a biproduct of fat degradation, indicating the age of a meat product. In this study, chicken breast samples were sliced into 1-cm-thick samples and stored for either 0, 3, 6, or 9 D at 4°C. Samples were imaged using a Vis-NIR HSI system (328–1,115 nm). Immediately after imaging, reference TBARS values were calculated using a traditional destructive extraction protocol and spectrophotometer to measure absorbance of the resulting filtrate at 532 nm. Using the hyperspectral images, a PLSR model was able to successfully predict TBARS values. Then, SPA was used in combination with PLSR to build a model based only on optimal wavelengths resulting in slightly worse prediction.

Another study to use a chemical measure used hydroxyproline as an indicator for meat tenderness of chicken breast fillets (Xiong et al., 2015c). Free-range and broiler chicken carcasses were acquired from a local market, from which strips of breast meat were cut. Hydroxyproline is a component of collagen, and meat high in hydroxyproline is correlated with low tenderness. After imaging using Vis-NIR HSI, hydroxyproline content was determined using traditional methods as reference values. Using regression coefficients to select optimal wavelengths and build a RC-PLSR model, hydroxyproline content could be successfully predicted. However, poultry is most commonly slaughtered at an age before collagen greatly affects meat tenderness (<8 wk), limiting the ability to predict tenderness using hydroxyproline (Coró et al., 2002). Because the age of the birds used in this study was not made explicit, it is not clear if using HSI to predict poultry tenderness in industry would be feasible.

The next 2 studies attempted to quantify TVB-N using Vis-NIR HSI as an indicator of spoilage (Khulal et al., 2016, Khulal et al., 2017). High TVB-N in meat products is correlated with high levels of microbial spoilage, making it usable as a quantitative spoilage index. In both studies, randomly selected samples were removed from refrigeration to determine reference TVB-N content and imaged every other day over the course of 9 D. Values of TVB-N over 15 mg/100 g were considered to be stale. By the final day, the range of TVB-N values was 25–43 mg/100 g. The main goal of this study was to determine if PCA or an ant colony optimization (ACO) was the best method for selecting optimal wavelengths to be used with back propagation artificial neural network for modeling TVB-N content. The study concludes that ACO has been underutilized in comparison to PCA, even though ACO is a superior method to PCA. In the following year, the same group repeated the study with the addition of fusing data from colorimetric sensors with HSI to predict TVB-N content. Fusing the data resulted in the PCA-back propagation artificial neural network model to have better prediction results than those determined by the previous study.

#### Sensory Attributes

Sensory attributes (e.g., springiness, tenderness, juiciness) of poultry products can be hard to quantify, as they frequently depend on the individual judgement of testers. However, HSI can be used to quantify the spectra of desirable sensory attributes to test if products meet standards demanded by consumers.

Springiness is the ability of meat to “bounce-back” when an external force is applied to it. A high level of springiness is desirable by consumers and is often a sensory indicator of fresh meat. In production lines, inspectors are trained to use the “finger method” to apply pressure to a poultry product and judge springiness. This is largely subjective, and not every sample can be tested. Xiong et al. (2014) attempted to quantify springiness of chicken breast fillet slices purchased at a local store using Vis-NIR HSI (Xiong et al., 2014). No information was provided on what time postmortem quality measurements were taken. After imaging, reference springiness values were calculated by twice-compression method using an Instron universal testing machine. Using a PLSR model based on selected optimal wavelengths by SPA, it was possible to accurately predict springiness.

The next attempt at quantifying sensory attributes was to detect “wooden breast condition” in chicken fillets (Yoon et al., 2016). Wooden breast condition is used to describe poultry meat with an uncharacteristically hard feel with no known cause. This study combined HSI data with data from optical coherence tomography (OCT). Chicken breast fillets of 8-week-old broilers were collected from a processing plant approximately 3 h postmortem. The exact time of imaging postmortem is not made explicit. OCT is only able to scan 1 cm^2^ at a time and is able to detect the texture associated with wooden breast condition. On the other hand, HSI can scan at a much quicker rate, but spectra from normal and wooden breast condition samples do not differ. However, HSI can be used to distinguish between fat, muscle tissue, and connective tissue. Because wooden breast condition is associated with fibrous connective tissue surrounding muscles (epimysium), HSI could be used to select regions of interest (i.e., epimysium) to be scanned by OCT. This study suggests that fusing the 2 technologies could potentially be done to increase the throughput time of OCT in future work.

The next study on sensory attributes proposes fusion of Vis-NIR HSI data and image textural features to classify tenderness by predicting shear force values of whole chicken breast fillets (Jiang et al., 2018a). Samples were prepared by deboning chicken breast fillets at 2, 4, or 24 h postmortem to obtain samples at a variety of tenderness levels. Traditional methods were used to measure Warner-Bratzler shear force reference values of samples. Combining textural and spectral data to build either PLS-DA or Radial basis function–support vector machine models resulted in high correct classification rates of tenderness compared to reference values.

The final study to quantify sensory attributes of poultry products also fused textural and spectral data, but this time to predict water-holding capacity of whole chicken breast fillets using NIR HSI (Yang et al., 2018b). Water-holding capacity is directly related to sensory attributes such as tenderness and juiciness. Quantification of water-holding capacity can be performed by measuring drip loss, expressible fluid, and salt-induced water gain. Samples were imaged approximately 2 h postmortem using HSI. This study used gray level co-occurrence matrix to obtain textural data of images. The PLSR model based on fusion of textural and spectral data performed the best for predicting drip loss and expressible fluid. However, spectral data alone performed better for salt-induced water gain predication.

## Conclusions

Since the first application to poultry products 20 yr ago, HSI has been shown to be a successful alternative to traditional screening methods. HSI can be applied to detect fecal contamination, food fraud, physical defects, microbiological contamination, and product quality. Traditional methods of detection are often tedious, destructive, not representative of the whole sample, or subject to the judgement of an inspector. In contrast, HSI has been shown to be capable of high-throughput online monitoring of poultry products. The following is a list of limitations that exist with applying HSI to poultry products and suggestions for future research:

• As with other applications, use of HSI is limited by the length of time needed to acquire, process, and classify images (Gowen et al., 2007). To date, only pushbroom and staredown modes of image acquisition have been used in imaging poultry products. Because snapshot imaging acquires images with one exposure and relies on less processing methods, it could increase the speed at which HSI could be applied (Hagan and Kudenov, 2013). The recent commercial availability of snapshot mode HSI systems could make snapshot imaging a reality for poultry products in future work. However, there are currently some limitations to snapshot systems. The main limitation of snapshot systems comes from the amount of data that must be collected and analyzed. Because of how large the data set behind each snapshot image is, it is only recently that technology capable of handling snapshot data fast enough has been developed (Hagan and Kudenov, 2013). Another limitation of snapshot systems is that they are typically more complex than scanning systems, requiring more advanced and expensive manufacturing methods (Hagan and Kudenov, 2013).
• Further limitations arise when HSI is applied to microbiology. For example, signal interference can occur from the high absorbance of water, cell motility can blur images, autoflorescence of biological samples can interact with illumination source, and heat from illumination can heat and alter or burn the sample (Gowen et al., 2015).
• Often samples are pretreated in unrealistic ways to prepare them for imaging (e.g., flattened, moisture is removed, using only one slice). However, these compromises are not unique to HSI. In addition to requiring sample pretreatment, traditional techniques are slow and often require sample destruction. In comparison with traditional techniques, the high speed and nondestructive feature of HSI makes it a worthwhile alternative.
• To date, HSI has been tested in-line with no physical barriers between the sample and the imaging system (Park et al., 2010, Yoon et al., 2011). This may be a limitation to accurately identifying the safety of products for consumers, as the classification is made before the final steps of production. Currently, after the product is imaged, it will likely go on to further packaging steps before it reaches the consumer. As the product encounters more surfaces after imaging, further opportunities arise for pathogens and spoilage bacteria to colonize (Rouger et al., 2017). Consequently, HSI should be implemented further down the production line in future work, once the products are completely processed and packaged, to ensure safe products for consumers. Although not the same as imaging through packaging, real-time HSI has been successfully applied to imaging meat through sausage casing (Feng et al., 2018). Future work is required to assess HSI utility for successfully imaging poultry products through packaging.

## References

[bib85] Boziaris I.S. (2014). Novel Food Preservation and Microbial Assessment Techniques.

[bib1] Castellini C., Berri C., Le Bihan-Duval E., Martino G. (2008). Qualitative attributes and consumer perception of organic and free-range poultry meat. Worlds. Poult. Sci. J..

[bib2] Centers for Disease Control and Prevention (CDC). (2015). Serotypes and the importance of serotyping *Salmonella*. https://www.cdc.gov/salmonella/reportspubs/salmonella-atlas/serotyping-importance.html.

[bib3] Chao K., Mehl P.M., Chen Y.R. (2002). Use of hyper-and multi-spectral imaging for detection of chicken skin tumors. Appl. Eng. Agric..

[bib4] Chao K., Yang C.C., Chen Y.R., Kim M.S., Chan D.E. (2007). Hyperspectral-multispectral line-scan imaging system for automated poultry carcass inspection applications for food safety. Poult. Sci..

[bib5] Chao K., Yang C.C., Kim M.S., Chan D.E. (2008). High throughput spectral imaging system for WHOLESOMENESS inspection OF chicken. Appl. Eng. Agric..

[bib6] Cho B.K., Chen Y.R., Kim M.S. (2007). Multispectral detection of organic residues on poultry processing plant equipment based on hyperspectral reflectance imaging technique. Comput. Electron. Agric..

[bib7] Cho B.K., Kim M.S., Chen Y.R. (2005). Hyperspectral imaging technique for detection of poultry fecal residues on food processing equipments ∗. Opt. Sensors Sens. Syst. Nat. Resour. Food Saf. Qual..

[bib8] Coró F.A.G., Youssef E.Y., Shimokomaki M. (2002). Age related changes in poultry breast meat collagen pyridinoline and texture. J. Food Biochem..

[bib9] Danezis G.P., Tsagkaris A.S., Camin F., Brusic V., Georgiou C.A. (2016). Food authentication: techniques, trends & emerging approaches.

[bib10] Du Z., Jeong M.K., Kong S.G. (2007). Band selection of hyperspectral images for automatic detection of poultry skin tumors. IEEE Trans. Autom. Sci. Eng..

[bib11] Eady M., Park B., Choi S. (2015). Rapid and early detection of Salmonella serotypes with hyperspectral microscopy and Multivariate data analysis. J. Food Prot..

[bib12] Eady M., Setia G., Park B. (2019). Detection of Salmonella from chicken rinsate with visible/near-infrared hyperspectral microscope imaging compared against RT-PCR. Talanta.

[bib13] EFSA, ECDC (2016). The European Union summary report on trends and sources of zoonoses, zoonotic agents and food?borne outbreaEFSA, ECDC, 2016. The European Union summary report on trends and sources of zoonoses, zoonotic agents and food?borne outbreaks in?2015. EFSA J. 14,. EFSA J..

[bib14] Elmasry G., Iqbal A., Sun D.W., Allen P., Ward P. (2010). Quality classification of cooked, sliced Turkey hams using NIR hyperspectral imaging system. J. Food Eng..

[bib15] Feng Y.Z., Elmasry G., Sun D.W., Scannell A.G.M., Walsh D., Morcy N. (2012). Near-infrared hyperspectral imaging and partial least squares regression for rapid and reagentless determination of Enterobacteriaceae on chicken fillets. Food Chem..

[bib16] Feng C.-H., Makino Y., Yoshimura M., Rodríguez-Pulido F.J. (2018). Real-time prediction of pre-cooked Japanese sausage color with different storage days using hyperspectral imaging. J. Sci. Food Agric..

[bib17] Feng Y.Z., Sun D.W. (2012). Determination of total viable count (TVC) in chicken breast fillets by near-infrared hyperspectral imaging and spectroscopic transforms. Talanta.

[bib18] Feng Y.Z., Sun D.W. (2013). Near-infrared hyperspectral imaging in tandem with partial least squares regression and genetic algorithm for non-destructive determination and visualization of Pseudomonas loads in chicken fillets. Talanta.

[bib19] Fisher M.E., Trampel D.W., Griffith R.W. (1998). Postmortem detection of acute septicemia in broilers. Avian Dis..

[bib20] Fletcher J.T., Kong S.G. (2003). Principal component analysis for poultry tumor inspection using hyperspectral fluorescence imaging. Proc. Int. Jt. Conf. Neural Networks.

[bib21] Garrido-Novell C., Garrido-Varo A., Pérez-Marín D., Guerrero J.E. (2018). Using spectral and textural data extracted from hyperspectral near infrared spectroscopy imaging to discriminate between processed pork, poultry and fish proteins. Chemom. Intell. Lab. Syst..

[bib22] Gowen A.A., Feng Y., Gaston E., Valdramidis V. (2015). Recent applications of hyperspectral imaging in microbiology. Talanta.

[bib23] Gowen A.A., O’Donnell C.P., Cullen P.J., Downey G., Frias J.M. (2007). Hyperspectral imaging – an emerging process analytical tool for food quality and safety control. Trends Food Sci. Technol..

[bib24] Hagan N., Kudenov M.W. (2013). Review of snapshot spectral imaging technologies. Opt. Eng..

[bib25] Heitschmidt G.W., Lanoue M., Mao C., May G. (1998). Hyperspectral analysis of fecal contamination: a case study of poultry, SPIE Conference on Pathogen Detection and Remediation for Safe Eating, Boston, MA.

[bib26] Heitschmidt G.W., Park B., Lawrence K.C., Windham W.R., Smith D.P. (2007). Improved hyperspectral imaging system for fecal detection on poultry carcasses. Trans. ASABE.

[bib27] Iqbal A., Sun D.W., Allen P. (2013). Prediction of moisture, color and pH in cooked, pre-sliced Turkey hams by NIR hyperspectral imaging system. J. Food Eng..

[bib28] Jia B., Yoon S.-C., Zhuang H., Wang W., Li C. (2017). Prediction of pH of fresh chicken breast fillets by VNIR hyperspectral imaging. J. Food Eng..

[bib29] Jiang H., Yoon S.-C., Zhuang H., Wang W., Lawrence K.C., Yang Y. (2018). Tenderness classification of fresh broiler breast fillets using visible and near-infrared hyperspectral imaging. Meat Sci..

[bib30] Jiang H., Yoon S.-C., Zhuang H., Wang W., Li Y., Lu C., Li N. (2018). Non-destructive assessment of final color and pH attributes of broiler breast fillets using visible and near-infrared hyperspectral imaging: a preliminary study. Infrared Phys. Technol..

[bib31] Jun W., Kim M.S., Cho B.K., Millner P., Chao K., Chan D.E. (2010). Microbial biofilm detection on food contact surfaces by macro-scale fluorescence imaging. J. Food Eng..

[bib32] Jun W., Kim M.S., Lee K., Millner P., Chao K. (2009). Assessment of bacterial biofilm on stainless steel by hyperspectral fluorescence imaging. Sens. Instrum. Food Qual. Saf..

[bib33] Jun W., Lee K., Millner P., Sharma M., Chao K., Kim M.S. (2008). Portable hyperspectral fluorescence imaging system for detection of biofilms on stainless steel surfaces. Page 698306 in Defense and Security 2008: Special Sessions on Food Safety. Proc. of SPIE - The International Society for Optical Engineering, Bellingham, WA.

[bib34] Kamruzzaman M., Makino Y., Oshita S. (2014). Rapid and non-destructive detection of chicken adulteration in minced beef using visible near-infrared hyperspectral imaging and machine learning. J. Food Eng..

[bib35] Kandpal L., Lee H., Kim M.S., Mo C., Cho B.K., Kandpal L.M. (2013). Hyperspectral reflectance imaging technique for visualization of moisture Distribution in cooked chicken breast. Sensors.

[bib36] Khulal U., Zhao J., Hu W., Chen Q. (2016). Nondestructive quantifying total volatile basic nitrogen (TVB-N) content in chicken using hyperspectral imaging (HSI) technique combined with different data dimension reduction algorithms. Food Chem..

[bib37] Khulal U., Zhao J., Hu W., Chen Q. (2017). Intelligent evaluation of total volatile basic nitrogen (TVB-N) content in chicken meat by an improved multiple level data fusion model. Sensors Actuators, B Chem..

[bib38] Kim T., Cho B.K., Kim M.S. (2010). Emission filter design to detect poultry skin tumors using fluorescence hyperspectral imaging. Rev. Colomb. Ciencias Pecu..

[bib39] Kim I., Kim M.S., Chen Y.R., Kong S.G. (2004). Detection of skin tumors on chicken carcasses using hyperspectral fluorescence imaging. Trans. ASAE.

[bib40] Kong S.G. (2003). Inspection of poultry skin tumor using hyperspectral fluorescence imaging. Int. Soc. Opt. Photon..

[bib41] Kong S.G., Chen Y.R., Kim I., Kim M.S. (2004). Analysis of hyperspectral fluorescence images for poultry skin tumor inspection. Appl. Opt..

[bib42] Lawlor J.B., Sheehan E.M., Delahunty C.M., Kerry J.P., Morrissey P.A. (2003). Sensory characteristics and consumer preference for cooked chicken breasts from organic, corn-fed, free-range and conventionally reared animals. Int. J. Poult. Sci..

[bib43] Lawrence K.C., Park B., Heitschmidt G.W., Windham W.R., Thai C.N. (2007). Evaluation of LED and tungsten-halogen lighting for fecal contaminant detection. Appl. Eng. Agric..

[bib44] Lawrence K.C., Windham W.R., Park B., Jeff Buhr R. (2003). A hyperspectral imaging system for identification of faecal and ingesta contamination on poultry carcasses. J. Near Infrared Spectrosc..

[bib45] Lawrence K.C., Windham W.R., Park B., Smith D.P., Poole G.H. (2004). Comparison between visible/NIR spectroscopy and hyperspectral imaging for detecting surface contaminants on poultry carcasses. Monit. Food Safety, Agric. Plant Heal..

[bib46] Lawrence K.C., Windham W.R., Smith D.P., Park B., Feldner P.W. (2006). Effect OF broiler carcass washing ON FECAL contaminant imaging. Trans. ASABE.

[bib47] Liu Y., Windham W.R., Lawrence K.C., Park B. (2003). Simple algorithms for the Classifcation of visible/near-infrared and hyperspectral imaging spectra of chicken skins, feces, and fecal contaminated skins. Appl. Spectrosc..

[bib48] Lu R., Chen Y.R. (1999). Hyperspectral imaging for safety inspection of food and agricultural products. Int. Soc. Opt. Photon..

[bib49] Nakariyakul S., Casasent D.P. (2004). Hyperspectral feature selection and fusion for detection of chicken skin tumors. Int. Soc. Opt. Photon..

[bib50] Nakariyakul S., Casasent D.P. (2009). Fast feature selection algorithm for poultry skin tumor detection in hyperspectral data. J. Food Eng..

[bib51] Oh M., Lee H., Torres I., Garrido-Varo A., Pérez-Marín D., Kim M.S. (2017). Analysis of pork and poultry meat and bone meal mixture using hyperspectral imaging. Int. Soc. Opt. Photon..

[bib52] Park B., Lawrence K.C., Windham W.R., Buhr R.J. (2002). Hyperspectral imaging for detecting fecal and ingesta contaminants on poultry carcasses. Trans. ASAE.

[bib53] Park B., Lawrence K.C., Windham W.R., Smith D.P. (2005). Detection of cecal contaminants in visceral cavity of broiler carcasses using hyperspectral imaging. Appl. Eng. Agric..

[bib54] Park B., Lawrence K.C., Windham W.R., Smith D.P. (2006). Performance of hyperspectral imaging system for poultry surface fecal contaminant detection. J. Food Eng..

[bib55] Park B., Lee S., Yoon S.-C., Sundaram J., Windham W.R., Hinton A., Lawrence K.C. (2011). AOTF hyperspectral microscopic imaging for foodborne pathogenic bacteria detection. Sens. Agric. Food Qual. Saf..

[bib56] Park B., Seo Y., Eady M., Yoon S.-C., Hinton A., Lawrence K.C., Gamble G. (2017). Classification of Salmonella serotypes with hyperspectral microscope imagery. Ann. Clin. Pathol..

[bib57] Park Y., Seo S.-C., Yoon A., Hinton, Windham W.R., Lawrence K.C. (2015). Hyperspectral microscope imaging methods to classify gram-positive and gram-negative foodborne pathogenic Bacteri Aerobic Campylobacter incubation View project poultry processing interventions View project. Trans. ASABE.

[bib58] Park B., Windham W.R., Lawrence K.C., Smith D.P. (2007). Contaminant classification of poultry hyperspectral imagery using a spectral Angle Mapper algorithm. Biosyst. Eng..

[bib59] Park B., Yoon S.-C., Lawrence K.C., Windham W.R. (2007). Fisher linear discriminant analysis for improving fecal detection accuracy with hyperspectral images. Trans. ASABE.

[bib60] Park B., Yoon S.-C., Windham W.R., Lawrence K.C., Kim M.S., Chao K. (2010). Line-scan hyperspectral imaging for real-time in-line poultry fecal detection. Sens. Instrum. Food Qual. Saf..

[bib61] Qin J., Chao K., Kim M.S., Kang S., Cho B.K., Jun W. (2011). Detection of organic residues on poultry processing equipment surfaces by LED-induced fluorescence imaging. Appl. Eng. Agric..

[bib62] Rouger A., Tresse O., Zagorec M. (2017). Bacterial contaminants of poultry meat: sources, species, and dynamics. Microorganisms.

[bib63] Smith D.P. (2001). Defects of pre- and post-deboned broiler breast. J. Appl. Poult. Res..

[bib86] United States Department of Agriculture. (1998). Poultry post-mortem inspection and reinspection: enforcing the zero tolerance for visible fecal material. Food Safety and Inspection Service, Directive 6150.1, Rev. 1..

[bib64] Wang H., Wang H., Liang L., Xu X.-L., Zhou G. (2018). Prevalence, genetic characterization and biofilm formation in vitro of staphylococcus aureus isolated from raw chicken meat at retail level in Nanjing, China. Food Control.

[bib65] Windham W.R., Heitschmidt G.W., Smith D.P., Berrang M.E. (2005). Detection of ingesta on pre-chilled broiler carcasses by hyperspectral imaging. Int. J. Poult. Sci..

[bib66] Windham W.R., Smith D.P., Berrang M.E., Lawrence K.C., Feldner P.W. (2005). Effectiveness of hyperspectral imaging system for detecting cecal contaminated broiler carcasses. Int. J. Poult. Sci..

[bib67] Windham W.R., Smith D.P., Park B., Lawrence K.C., Feldner P.W. (2003). Algorithm development with visible/near-infrared spectra for detection of poultry feces and ingesta. Trans. ASAE.

[bib68] Wu D., Sun D.W. (2013). Advanced applications of hyperspectral imaging technology for food quality and safety analysis and assessment: a review - Part I: Fundamentals. Innov. Food Sci. Emerg. Technol..

[bib69] Xiong Z., Sun D.W., Dai Q., Han Z., Zeng X.A., Wang L. (2014). Application of visible hyperspectral imaging for prediction of springiness of fresh chicken meat. Food Anal. Methods.

[bib70] Xiong Z., Sun D.W., Pu H., Xie A., Han Z., Luo M. (2015). Non-destructive prediction of thiobarbituric acid reactive substances (TBARS) value for freshness evaluation of chicken meat using hyperspectral imaging. Food Chem..

[bib71] Xiong Z., Sun D.W., Pu H., Zhu Z., Luo M. (2015). Combination of spectra and texture data of hyperspectral imaging for differentiating between free-range and broiler chicken meats. LWT - Food Sci. Technol..

[bib72] Xiong Z., Sun D.W., Xie A., Han Z., Wang L. (2015). Potential of hyperspectral imaging for rapid prediction of hydroxyproline content in chicken meat. Food Chem..

[bib73] Yang C.C., Chao K., Kim M.S. (2009). Machine vision system for online inspection of freshly slaughtered chickens. Sens. Instrum. Food Qual. Saf..

[bib74] Yang Y., Wang W., Yoon S.-C., Zhuang H., Jiang H., Jia B. (2018). Prediction of quality traits of chicken breast fillets by different spectral range of hyperspectral imaging Yi. Am. Soc. Agric. Biol. Eng..

[bib75] Yang Y., Wang W., Zhuang H., Yoon S.-C., Jiang H. (2018). Fusion of spectra and texture data of hyperspectral imaging for the prediction of the water-holding capacity of fresh chicken breast Filets. Appl. Sci..

[bib76] Ye X., Iino K., Zhang S. (2016). Monitoring of bacterial contamination on chicken meat surface using a novel narrowband spectral index derived from hyperspectral imagery data. Meat Sci..

[bib77] Yoon S.-C., Bowker B.C., Zhuang H. (2016). Toward a fusion of optical coherence tomography and hyperspectral imaging for poultry meat quality assessment. IS T Int. Symp. Electron. Imaging Sci. Technol..

[bib78] Yoon S.-C., Lawrence K.C., Line J.E., Siragusa G.R., Feldner P.W., Park B., Windham W.R. (2010). Detection of Campylobacter colonies using hyperspectral imaging. Sens. Instrum. Food Qual. Saf..

[bib79] Yoon S.-C., Lawrence K.C., Siragusa G.R., Line J.E., Park B., Feldner P.W. (2009). Hyperspectral reflectance imaging for detecting a foodborne pathogen: Campylobacter. Trans. Am. Soc. Agric. Biol. Eng..

[bib81] Yoon S.-C., Lawrence K.C., Smith D.P., Park B., Windham W.R. (2008). Embedded bone fragment detection in chicken fillets using transmittance image enhancement and hyperspectral reflectance imaging. Sens. Instrum. Food Qual. Saf..

[bib82] Yoon S.-C., Park B., Lawrence K.C., Windham W.R., Heitschmidt G.W. (2011). Line-scan hyperspectral imaging system for real-time inspection of poultry carcasses with fecal material and ingesta. Comput. Electron. Agric..

[bib83] Yoon S.-C., Windham W.R., Ladely S., Heitschmidt G.W., Lawrence K.C., Park B., Narang N., Cray W.C. (2012). Hyperspectral imaging for detection of non-O157 Shiga-toxin producing Escherichia coli (STEC) serogroups on spread plates of mixed cultures. Sens. Agric. Food Qual. Saf. IV.

